# Supplementary material for: Factors associated with contraceptive use among reproductive-age women during a pandemic: Evidence from a small developing state
Source: PLOS Glob Public Health. 2026 May 22;6(5):e0006049. doi: 10.1371/journal.pgph.0006049 (PMC13196957; doi:10.1371/journal.pgph.0006049)
Supplement: S1 Table — (DOCX) [file pgph.0006049.s001.docx]

S1 Table: Sensitivity analysis

| **Variable** | **MI model OR (95% CI)** | **Complete-case OR (95% CI)** |
| --- | --- | --- |
| HADS anxiety score | 1.06 (1.00–1.12) | 1.06 (1.04–1.08) |
| HADS depression score | 0.98 (0.92–1.05) | 0.97 (0.95–0.99) |
| Marijuana use | 1.27 (0.80–2.03) | 1.02 (0.88–1.18) |
| Alcohol use (level 2) | 1.22 (0.85–1.76) | 0.94 (0.84–1.05) |
| Alcohol use (level 3) | 0.97 (0.61–1.54) | 0.65 (0.56–0.76) |
| Alcohol use (level 4) | 1.53 (0.95–2.46) | 1.30 (1.13–1.50) |
| Breadwinner category 2 | 1.26 (0.76–2.08) | 1.21 (1.05–1.41) |
| Breadwinner category 3 | 1.19 (0.56–2.53) | 1.27 (0.99–1.64) |
| Breadwinner category 4 | 0.63 (0.31–1.29) | 0.33 (0.25–0.44) |
| Breadwinner category 5 | 0.74 (0.48–1.12) | 0.76 (0.67–0.86) |
| Tertiary education | 1.68 (1.24–2.28) | 1.28 (1.17–1.41) |
| Partner not cohabiting | 1.57 (1.10–2.22) | 1.42 (1.26–1.59) |
| Married/cohabiting | 2.49 (1.65–3.77) | 2.64 (2.32–3.01) |
| Separated/divorced/widowed | 0.90 (0.34–2.35) | 0.68 (0.46–0.99) |
| Age (per year) | 0.98 (0.95–1.00) | 0.98 (0.97–0.99) |
