# Supplementary material for: Factors associated with contraceptive use among reproductive-age women during a pandemic: Evidence from a small developing state
Source: PLOS Glob Public Health. 2026 May 22;6(5):e0006049. doi: 10.1371/journal.pgph.0006049 (PMC13196957; doi:10.1371/journal.pgph.0006049)
Supplement: Attachment — Submitted filename: Reviews.pdf [file pgph.0006049.s002.pdf]

Comments on “Factors associated with the contraceptive use among reproductive-age women during a pandemic: Evidence from a small developing state.

First of all, I would like to thank all the authors for conducting research on such sensitive and often neglected issues, especially at the time of health emergencies. You have done such good work. Here are some of my reviews to help strengthen and support your study. These are my views and you have every right to disagree with it.

Abstract

Results: The abstract says that logistic analysis was performed but only p-values are reported. For logistic regression, reporting odds ratios with 95% Confidence Intervals is necessary to interpret the magnitude and direction of association. Again, Variables, alcohol and marijuana use had >15% missing data which limited? Limited what? The language is very unclear and this whole makes it confusing for the readers to understand.

Introduction

Line 39,40,41: “During the early phase of the COVID-19 pandemic, disruptions to health systems worldwide substantially reduced access to SRH services, with women disproportionately affected….”

Comment: The authors have used the 4 references for a sentence that seems a bit overcrowded. Do this all articles say the same? Or the other were proving the point of their sentence than providing the reference?

Line 50: “systems operate under markedly different structural constraints” What kind of structural constraints? It would have been more informative If the types of structural constraints are mentioned

Line 53,54: “Legislative and policy reforms have strengthened protections for women’s and children’s health; however, implementation gaps persist”. What were the previous legislative policies, and what are the new ones that have helped in strengthening the protection for women’s and children’s health? I think an example would provide a context and help readers grasp the underlying issues.

Line 64,65: “data on funding and service provision for gender-based violence prevention and response are limited”

I think this sentence needs a bit more clarity, authors have mentioned about the provision for GBV but does this GBV explicitly refer to SRH or other domestic issues?

Methods

Line 146 to154: Same repetition of the question for assessing the contraceptive use, again the repetition of age-group.

Line 182 the table description can be kept as in a single column such as; n(%) 237(21.6%).

Line 184: "Percentages may not add up to 100 due to rounding…. I think this can be kept as a footnote such as, *note: percentages may not add up to 100 due to rounding.* This helps the reader.

"This study explores the core topic and reflects strong analytical capacity, particularly for this large sample size and clear methods description. While the use of Univariate Logistic Regression to assess the association is appropriate for your objective, reporting of just p-values limits the interpretation of the findings. Presenting odds ratios with 95% CI would help enhance transparency and allow readers to better understand the magnitude and direction of association. Also, if the objective was only to check the association, then chi-square alone would have been sufficient. While I understand that everyone has their own way of doing the research study but it would be great if we do what's specifically a statistical analyses are needed for, the authors have made methodologically validated their choice of univariable logistic regression, logistic regression is primarily intended for estimating the odds ratios and effect sizes, rather than solely for testing associations. For simple associations between categorical variables, Chi-square would have been sufficient. Using logistic regression without reporting the odds limits the study's potential to yield a meaningful result. Nevertheless, the approach is understandable."

Discussion

Although the discussion provides great details, it felt more like a thematic analysis than the standard discussion.

This is a very good and informative study.

Good luck!
