# Supplementary material for: Factors associated with contraceptive use among reproductive-age women during a pandemic: Evidence from a small developing state
Source: PLOS Glob Public Health. 2026 May 22;6(5):e0006049. doi: 10.1371/journal.pgph.0006049 (PMC13196957; doi:10.1371/journal.pgph.0006049)
Supplement: Attachment — Submitted filename: Response to the Editor and Reviewers.docx [file pgph.0006049.s003.docx]

Response to the Editor and Reviewers

Manuscript Title: Factors associated with contraceptive use among reproductive-age women during a pandemic: Evidence from a small developing state

Manuscript ID: PGPH-D-26-00542

Dear Editor and Reviewers,

We sincerely thank you and the reviewers for the careful evaluation of our manuscript and for the thoughtful, constructive feedback. We appreciate the recognition of the importance of this work, particularly in the context of health emergencies. We have revised the manuscript extensively in response to the comments and believe it is now significantly strengthened.

Below, we provide a detailed, point-by-point response. Reviewer comments are presented in italic, followed by our responses.

**Reviewer #1 Comments and Author Responses**

Abstract

Comment 1:

*“The abstract says that logistic analysis was performed but only p-values are reported. For logistic regression, reporting odds ratios with 95% Confidence Intervals is necessary…”*

Response:

Thank you for this important observation. We agree that reporting odds ratios (ORs) with 95% confidence intervals enhances interpretability. We have revised the abstract to include ORs and corresponding 95% CIs for key variables.

Revision made:

use. Moderate-to-severe anxiety symptoms were reported by 36.5%, and 39.0% reported moderate or clinically significant global distress. Sensitivity analyses comparing the multiple imputed models with a complete-case logistic regression model produced similar results. Tertiary education (OR 1.68; 95 %CI 1.24 - 2.28), partnered and not cohabitating (OR 1.57; 95% CI 1.10 - 2.22) married and cohabitating (OR 2.49; 95% CI 1.65 - 3.77) were most significantly associated with contraceptive use. There were also positive associations between contraceptive use and HADS anxiety (OR 1.06, 95% CI (1.00 - 1.12) and age (per year) (OR 0.98; 95% CI (0.95 – 1.00).

(Page 3, Lines 25-32)

Comment 2:

*“Variables, alcohol and marijuana use had >15% missing data which limited? … language is unclear.”*

Response:

We appreciate this clarification. We have revised the sentence to explicitly state how missing data affected the analysis.

Revision made:

Further, variables with incomplete observations (including alcohol and marijuana use and items in the HADS) were imputed. Sensitivity analyses were conducted to assess the robustness of the findings. Regression model estimated using multiple imputed data sets were compared with complete-case analyses restricted to observations without missing values. Results were consistent across these models.

(Page 2, Lines 14-19)

Introduction

Comment 3:

*“Multiple references used for one sentence… are they all supporting the same point?”*

Response:

Thank you for highlighting this. We have streamlined the references to ensure each citation clearly supports a distinct aspect of the statement.

Revision made:

Removed redundant/less pertinent citations

(Page 4-23)

Comment 4:

*“What kind of structural constraints?”*

Response:

We agree that specificity improves clarity. We have added examples of structural constraints relevant to the study setting.

Revision made:

Regional analyses frequently aggregate Caribbean countries with Latin America, masking heterogeneity and obscuring the specific needs of smaller island nations. In these settings, health systems are shaped by different structural constraints, including limited economies of scale due to small population sizes, persistent health workforce shortages, geographic isolation, dependence on imported medical supplies, and heightened vulnerability to economic instability and climate-related disasters.

(Page 4, Lines 59-65)

Comment 5:

*“Legislative and policy reforms… examples needed.”*

Response:

Thank you for this suggestion. We have included specific examples of legislative and policy reforms to provide context.

Revision made:

For example, earlier legislative and policy environments in several Caribbean countries limited adolescents’ access to sexual and reproductive health (SRH) services and, in some cases, resulted in the exclusion of pregnant adolescents from formal education. Recent reforms have sought to address these gaps through the introduction of adolescent-responsive SRH policies, including the application of competency-based guidelines (e.g., Gillick competence), the implementation of school re-entry policies for pregnant adolescents, and the expansion of legal protections against gender-based violence. Collectively, these reforms have strengthened access to essential services and enhanced protections for women and children across the region however, implementation gaps persist (7). These gaps are driven by limited resources, workforce constraints, and uneven translation of policy into service delivery (6, 8).

(Page 5, Lines 69-79)

Comment 6:

*“Clarify whether GBV refers to SRH or broader domestic issues.”*

Response:

We appreciate this point. We have clarified that gender-based violence (GBV) in this context refers to a broader context.

Revision made:

data on funding and service provision including gender-based violence, broadly encompassing intimate partner, domestic, and sexual violence, prevention and response are limited

(Page 5, Lines 88-90)

Methods

Comment 7:

*“Repetition of contraceptive use question and age-group.”*

Response:

Thank you for identifying this redundancy. We have removed repeated descriptions to improve clarity and conciseness.

Comment 8:

*“Table formatting (n(%)) suggestion.”*

Response:

Thank you for this suggestion. While presenting values as n (%) in a single column can be concise, we found that this format resulted in visually dense tables that were more difficult to read, particularly given the number of variables presented. We have therefore retained separate columns for counts and percentages to enhance clarity and accessibility for readers. We will, of course, adhere to any formatting preferences required by the journal during typesetting.

Comment 9:

*“Footnote for rounding percentages.”*

Response:

We agree and have moved this statement to a table footnote.

Revision made:

(Table 1, Note:

^a.^ Percentages may not sum to 100 due to rounding.

^b.^Contraceptive use assessed among non-pregnant women only.

Comment 10 (Statistical Analysis):

*“Use of logistic regression but only p-values reported… chi-square may have been sufficient… logistic regression should report odds ratios.”*

Response:

Thank you for this important observation. We agree that logistic regression is most informative when effect estimates are presented as odds ratios (ORs) with 95% confidence intervals (CIs). In the original submission, univariable analyses were used primarily as a screening step to identify variables for inclusion in the multivariable model, and therefore only p-values were reported.

The primary inferential analyses in this study are based on multivariable logistic regression models conducted after multiple imputation to address missing data, for which ORs and 95% CIs are now reported.

To improve transparency and align with best practices, we have now revised the manuscript to include ORs and 95% CIs for the univariable analyses as well.

Revision made:

Table 2. Univariable Associations Between Selected Factors and Current Contraceptive Use

Severe HADS anxiety scores were associated with higher odds of contraceptive use (OR 1.51; 95% CI 1.05-2.16), indicating greater likelihood of use compared with the reference group. ~~Severe HADS anxiety scores were associated with contraceptive use (OR 1.51; 95% CI 1.05 -~~ 2.16~~)., with higher-anxiety respondents demonstrating greater likelihood of contraceptive use compared to the reference category~~ . Similarly, clinically significant HADS global distress was significantly associated with contraceptive use (OR 1.54; 95% CI 1.04-2.29). In contrast, HADS depressive symptoms alone were not significantly associated with contraceptive use ~~(p=0.211).~~

Sociodemographic and relationship correlates

Relationship status was strongly associated with contraceptive use. Compared with single women, those who were partnered but not cohabitating were more likely to use contraception (OR 1.54, 95%CI 1.10-2.16), and those married or cohabitating showed a stronger association (OR 2.52; 95% CI 1.80-3.52). No association was observed among women who were separated, divorced, or widowed ~~(p=0.49).~~

Education also showed a strong association: women with university education were significantly more likely to report contraceptive use than those with lower educational attainment (OR 1.64; 95% CI 1.24-2.17).

Age was associated with contraceptive use, with women aged 26–39 having a statistically significant association with contraceptive use compared to women aged 40–49 years, while women aged 18–25 years having no statistical significance.

Multivariate analysis

Logistic regression model estimates using 20 multiple imputed datasets indicated that contraceptive use was significantly associated with education, relationship status and age. Table 3 shows that individuals with tertiary education had higher odds or contraceptive use compared to those without tertiary education (OR 1.68; 95% CI: 1.24-2.28). Compared to single individuals, those in partnerships but non-cohabitating (OR 1.57; 95% CI 1.10-2.22) and those who were married or cohabitated (OR 2.49, 95% CI 1.65-3.77) had significantly greater odds of contraceptive use. In bivariate analyses, women aged 26–32 and 33–39 differed significantly in contraceptive use compared to those aged 40–49, while no significant difference was observed for women aged 18–25. In multivariable analysis treating age as a continuous variable, increasing age was associated with a modest reduction in contraceptive use (OR = 0.98 per year, 95% CI: 0.95–1.00). Anxiety symptoms showed a marginal positive association with contraceptive use (OR 1.06, per unit increase, p=0.066), whereas depressive symptoms, marijuana consumption and breadwinner status were not significantly associated with contraceptive use. Figure 1 shows a forest-plot of the adjusted odds ratios.

Sensitivity analyses comparing the imputed model with a complete-case logistic regression showed similar results. Differences between models were primarily reflected in narrower confidence intervals in the complete-case analysis (see supplementary Table 1).

Table 3. Multivariate Associations Between Selected Factors and Current Contraceptive Use based on MICE.

(Page 15-20, Lines 235-312; Tables 2 and 3)

Discussion

*Comment 11:*

*“Discussion felt more like a thematic analysis than a standard discussion.”*

Response:

We appreciate this insightful feedback. We have revised the discussion to align more closely with conventional structure by:

Focusing on key findings rather than thematic expansion

Clearly linking results to existing literature

Reducing descriptive narrative elements

Strengthening interpretation and public health implications

Revision made:

We restructured the paragraphs and removed some the headings to reflect traditional analytic style.

(Page 21-24, Lines 312–391)

**Reviewer #2: Comments and Author Responses**

Comment 1 (Statistical approach and reporting):

*“Discrepancy between methods and results… logistic regression used but only p-values reported… why not chi-square… logistic regression should estimate odds ratios…”*

Response:

We thank the reviewer for this thoughtful and detailed comment. We agree that logistic regression is most informative when effect estimates are presented as odds ratios (ORs) with 95% confidence intervals (CIs), and we appreciate the opportunity to clarify and strengthen our approach.

In the original submission, univariable logistic regression analyses were used primarily as a preliminary screening step, and only p-values were reported for brevity. However, we acknowledge that this limited interpretability and may have created the impression of inconsistency between methods and results.

In response, we have revised the manuscript to:

Report ORs and 95% CIs for univariable logistic regression analyses

Clarify the rationale for using logistic regression rather than chi-square tests, emphasizing its ability to estimate effect size and direction

Explicitly distinguish between exploratory (univariable) and primary inferential (multivariable) analyses

Importantly, the primary findings of this study are based on multivariable logistic regression models conducted after multiple imputation to address missing data, which provide adjusted and methodologically robust effect estimates.

These revisions improve clarity, transparency, and alignment with standard statistical reporting practices.

Comment 2 (Clarification of GBV in Introduction):

*“Introduction sections should clarify Gender Based Violence in terms of SRH too”*

Response:

Thank you for this suggestion. We have revised the Introduction to clarify that gender-based violence (GBV) is used in a broad sense within the context of sexual and reproductive health (SRH), encompassing intimate partner, domestic, and sexual violence.

Revision made:

domestic financing for HIV and reproductive health programs, data on funding and service provision including gender-based violence, broadly encompassing intimate partner, domestic, and sexual violence, prevention and response are limited (9 ).

(Page 5, Lines 88–90)

Comment 3 (Discussion resembles thematic analysis):

*“Discussion felt more like a thematic analysis… were themes created or based on data collection tools?”*

Response:

We appreciate this insightful observation. The discussion was not based on a formal qualitative thematic analysis; rather, the structure reflected an attempt to organize findings across key domains measured in the survey instrument.

To improve clarity and align with conventional quantitative reporting:

We have restructured the Discussion to focus on key findings rather than thematic groupings

Removed implicit thematic framing

Strengthened linkage between results and existing literature

This revision ensures the discussion more clearly reflects the quantitative nature of the study.

**Reviewer #3: Comments and Author Responses**

Comment 1 (Data availability and public access):

*Data not fully available; only available upon request*

Response:

We thank the reviewer for highlighting this important requirement. In accordance with the journal’s data sharing policy, we have now deposited the dataset in a publicly accessible repository.

Specifically:

The dataset is now available via Figshare

A DOI has been assigned to ensure accessibility and citability

We have updated the Data Availability Statement accordingly to reflect unrestricted access to the data.

Revision made:

The raw data underlying this study have been deposited in a publicly accessible repository on Figshare and are available at https://doi.org/10.6084/m9.figshare.32099512.

The dataset is currently under embargo and will be made publicly available without restriction upon publication.

(Page 3, Lines 41-44)

Comment 2 (Univariable analysis and lack of multivariable modeling):

*Concern that only univariable models were used and confounding not addressed*

Response:

We appreciate this important clarification. While the initial submission emphasized univariable analyses, the study does include multivariable logistic regression models.

To improve clarity:

We have strengthened the description of multivariable analyses in the Methods section

Clearly presented adjusted ORs and 95% CIs in the Results

Emphasized that multivariable models were conducted using multiply imputed datasets to address missing data

These revisions ensure that the manuscript accurately reflects the analytical approach and addresses concerns regarding confounding.

Comment 3 (Cross-sectional design and exploratory nature):

*Limitations regarding causality, temporality, and exploratory nature*

Response:

We agree with the reviewer and appreciate the thoughtful summary of these limitations. These points are consistent with the intended scope of the study.

We have:

Strengthened the limitations section to explicitly emphasize the cross-sectional design and lack of causal inference

Reinforced that findings should be interpreted as exploratory and hypothesis-generating

Comment 4 (Missing data and variable inclusion):

*Concerns regarding variables exceeding missing data threshold*

Response:

Thank you for this important observation. We have clarified in the Methods and Results sections that:

Variables with substantial missing data (e.g., alcohol and marijuana use) were retained due to conceptual relevance

These variables were handled using multiple imputation

Findings related to these variables are interpreted cautiously

This clarification improves transparency and aligns interpretation with data quality considerations.

**Reviewer #4: Comments and Author Responses**

Comment (Data repository compliance):

*Request for confirmation of data deposition and repository details*

Response:

We thank the reviewer for this important comment. In compliance with journal requirements, we have deposited all relevant raw data in a publicly accessible repository.

Repository: Figshare

DOI: https://doi.org/10.6084/m9.figshare.32099512

The Data Availability Statement has been revised to include:

Repository name

Persistent identifier (DOI)

Confirmation of unrestricted access (once accepted for publication)

These changes ensure transparency, reproducibility, and adherence to journal standards.

We are grateful for the reviewer’s thoughtful and constructive comments, which have significantly improved the clarity, rigor, and overall quality of our manuscript. We hope that the revisions meet expectations and we would be pleased to make any further modifications if required.

Thank you for your consideration.

Sincerely,

Clemon George

On behalf of all authors
